# Antimicrobial Properties and Mode of Action of Cryptdin-4, a Mouse α-Defensin Regulated by Peptide Redox Structures and Bacterial Cultivation Conditions

**DOI:** 10.3390/antibiotics12061047

**Published:** 2023-06-14

**Authors:** Yi Wang, Yuchi Song, Shaonan Yan, Rina Hiramine, Yuki Ohnishi, Yuki Yokoi, Kiminori Nakamura, Takashi Kikukawa, Tokiyoshi Ayabe, Tomoyasu Aizawa

**Affiliations:** 1 Laboratory of Protein Science, Graduate School of Life Science, Hokkaido University, Sapporo 060-0810, Japan; 2 Innate Immunity Laboratory, Graduate School of Life Science, Hokkaido University, Sapporo 060-0808, Japan; 3 Laboratory of Biological Information Analysis Science, Graduate School of Life Science, Hokkaido University, Sapporo 060-0808, Japan

**Keywords:** antimicrobial peptide, defensin, membrane disruption, oxidative stress, *Escherichia coli*

## Abstract

Cryptdin-4 (crp4) is an enteric α-defensin derived from mice, and is a main mediator of immunity to oral infections and a determinant of the composition of the intestinal microbiota. Structurally, crp4 exists in two states: the oxidized form (crp4oxi), constrained by three invariant disulfide bonds, and the reduced form (crp4red) with six free thiol groups, both of which exist in the intestinal tract. In this study, the antibacterial mechanisms of crp4 in both forms under aerobic and anaerobic conditions were investigated using *Escherichia coli* (*E. coli*), an anaerobic facultative bacterium, as a model. Fluorescent dye studies revealed that both crp4oxi and crp4red exhibited antimicrobial activity against cells cultured under aerobic conditions via rapid membrane depolarization. Furthermore, the antioxidant treatment experiments suggested that only crp4oxi exhibited antimicrobial activity by the induction and accumulation of reactive oxygen species (ROS). However, under anaerobic culture conditions, the ability of both forms to disrupt the function of bacterial membranes decreased and activity was greatly reduced, but crp4red maintained some antimicrobial activity. This activity may be due to the inhibition of intracellular functions by DNA binding. Altogether, these data indicate that, according to its redox structure and the environmental redox conditions, crp4 could perform different antimicrobial activities via different mechanisms.

## 1. Introduction

Antimicrobial peptides (AMPs), a diverse family of short peptides that are important for plants and animals, are powerful weapons in host defense mechanisms and are present in all life domains [1]. Defensins, which are widely generated by fungi, insects, and vertebrates, are endogenous cationic AMPs that act as the main effectors of the innate immune system, owing to their broad-spectrum antimicrobial activities [2]. α-Defensins in the mammalian intestinal tract are expressed in the granules of Paneth cells, secreted into the lumen of the small intestinal crypts, and are called cryptdins (crps) in mice [3,4]. Among cryptdin family members, cryptdin-4 (crp4) has the highest microbicidal activity and contributes to innate immunity in the mouse intestine [5]. Structurally, crp4, with a molecular weight of approximately 4.5 kDa, consists of a three-stranded sheet structure, formed with the aid of three paired disulfide bonds, namely, Cys 1–Cys 6, Cys 2–Cys 4, and Cys 3–Cys 5 [6]. In addition to the oxidized form of crp4 (crp4oxi), the disulfide-null reduced-form crp4 (crp4red) has been observed in the reducing environment of the intestinal tract, and its physiological role is attracting attention [5,7,8]. Crp4 was found to show selective bactericidal activity against intestinal microbiota, which is dependent on disulfide bonds. Crp4oxi and crp4red have also been detected in feces and are associated with homeostasis and dysbiosis of the intestinal microbiota. Therefore, the antimicrobial activity of crp4s in the entire intestinal environment, including the colon and small intestine, has attracted attention. However, the antibacterial mechanism contributing to the bacteria-killing selectivity is poorly understood.

Studies on the mode of action (MOA) of AMPs are generally conducted under standard culture conditions for the target bacterial species, and may not necessarily reflect the conditions that occur in vivo, such as the oxygen gradient in the natural niche of the intestinal tract [9,10]. It has been recently discovered that AMPs exhibit different antimicrobial activities and mechanisms, depending on their environment. For instance, short peptides, such as LL-37, Melittin, and CM15, exhibit significantly higher killing activity against *E. coli* under aerobic conditions than under anaerobic conditions through multiple actions, such as membrane perturbation, induction of strong oxidative stress, and intracellular effects [11,12,13]. Additionally, a series of antimicrobial peptides with different redox states, including crp4, exhibit different activities. For example, the activity of human-defensin 1 (hBD1) is significantly increased when its disulfide bonds are reduced. The reduced type provide broad protection by entrapping bacteria in extracellular net structures, thereby preventing bacterial invasion. However, oxidized hBD1 is concentrated in the periplasm of Gram-negative bacteria, leading to bleb formation and bacterial death [14,15].

Unfortunately, most existing reports on crp4 focus on the assessment of antibacterial activities against pathogenic bacteria in vitro [5,16], which have been mainly tested under standard experimental conditions that do not necessarily reflect local conditions in vivo. Within the small intestine, the oxygen concentration decreases in the order of the duodenum, jejunum, and ileum, and becomes highly anaerobic in the colon. Even in the same region of the intestine, there is a steep oxygen gradient, from high oxygen concentrations near the epithelial surface to severe anaerobic conditions in the center of the lumen [17,18]. With this background, it is very important to investigate the mechanism of action of oxidized and reduced forms of crp4 (crp4oxi and crp4red) against the same bacteria under aerobic and anaerobic growth conditions, as much remains unknown about their mechanism of action. Therefore, this study compared the antimicrobial activity of crp4oxi and crp4red and their MOA under aerobic and anaerobic culture conditions using *Escherichia coli* (*E. coli*), which is a facultatively anaerobic commensal bacteria of the colon.

## 2. Results

### 2.1. Comparing the Activities of Crp4oxi and Crp4red against E. coli Cultured under Aerobic and Anaerobic Conditions

**Figure 1:**
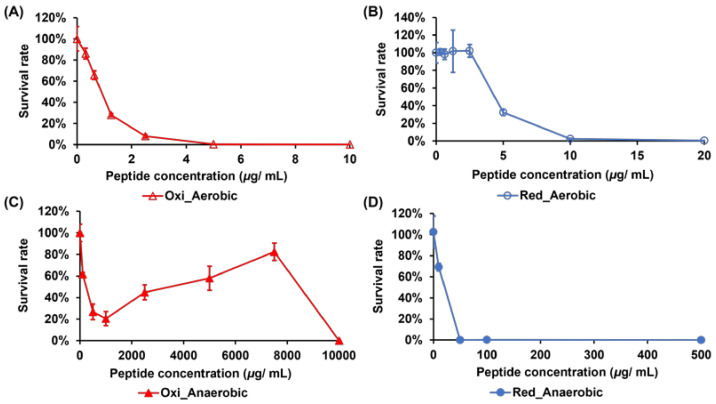
Bactericidal activity of crp4oxi (**A**,**C**) and crp4red (**B**,**D**) against *E. coli* under aerobic and anaerobic culture conditions, respectively. The bacterial concentration was 1 × 10^7^ CFU/mL. The data shown reflect the mean ± SEM of six replicates.

To assess the antibacterial activity of crp4oxi and crp4red, the minimum bactericidal concentration (MBC) was determined using the *E. coli* K12 strain, which was cultivated under aerobic or anaerobic conditions (Figure 1). MBC was defined as the concentration of the antimicrobial agent at which no visible colonies were observed on the plate. Under aerobic conditions, both crp4oxi and crp4red showed high antimicrobial activity. Crp4oxi (Figure 1A) and crp4red (Figure 1B) showed MBCs of 5 and 10 μg/mL, respectively, which is consistent with previous reports on their efficient antibacterial activities [5,16]. In contrast, the antimicrobial activities of both peptides were inhibited under anaerobic conditions (Figure 1C,D). First, the activity of crp4oxi was dramatically weaker, with approximately 20% survival, even at concentrations reaching 1000 μg/mL. At even higher concentrations, the activity weakened further; however, at 10,000 μg/mL, *E. coli* was completely killed. This suppression of activity at higher antimicrobial peptide concentrations may be related to a phenomenon occasionally reported for aggregating peptides [19,20]. The MBC of crp4red was 100 μg/mL under anaerobic conditions, which was approximately five times higher than that under aerobic conditions, also confirming a decrease in antimicrobial activity, although not as extreme as that of crp4oxi. Our results suggest that, depending on the redox structures of crp4 and the oxygen environment, they exhibit different antimicrobial activities. In particular, crp4oxi showed almost no activity under anaerobic conditions, but extremely strong activity under aerobic conditions, indicating that the presence of oxygen might be an important factor in activating its bactericidal performance.

### 2.2. Evaluation of Outer and Inner Membrane Damages by Fluorescence Measurements

To investigate the differences in the activities of crp4oxi and crp4red under aerobic and anaerobic conditions in detail, we first investigated one of the most common mechanisms of antimicrobial peptide activity: membrane damage. Two types of fluorescence experiments were performed: N-Phenylnaphthalen-1-amine (NPN), a hydrophobic fluorescent probe that fluoresces weakly in an aqueous environment, but strongly when it enters the interior of the outer membrane [21]. The inner membrane depolarization is monitored via the fluorescent signal generated by the efflux of a self-quenching marker, 3,3′-dipropylthiadicarbocyanine iodide (DiSC_3_-5) [22].

**Figure 2:**
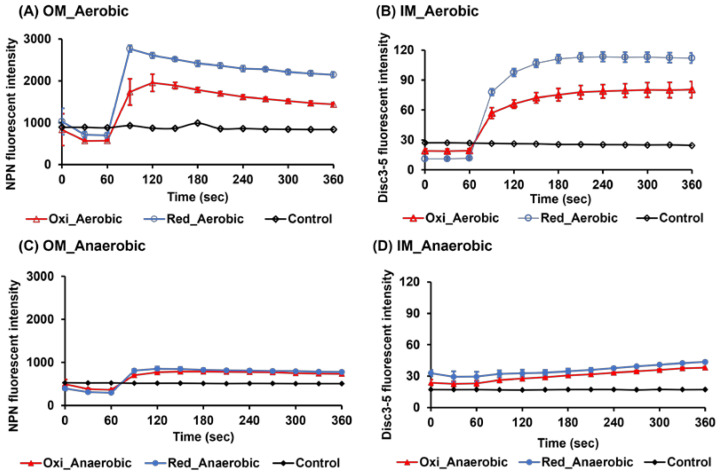
The effect of crp4oxi and crp4red on the membrane permeabilization of *E. coli* under aerobic and anaerobic cultivation. Outer membrane (OM) (**A**,**C**) and inner membrane (IM) (**B**,**D**) permeabilization were detected by the NPN uptake assay and Disc-5 fluorescent experiment, respectively. The bacterial concentration was 4 × 10^7^ CFU/ mL, and the peptide concentration was 80 μg/mL. Bacterial cells were without peptide treatment as control. The data shown reflect the mean ± SEM of three replicates.

Figure 2A,B show the changes in fluorescence intensity when the peptides were added at 80 μg/mL above their respective MBCs in *E. coli* under aerobic culture conditions. This concentration was chosen to simultaneously examine the effects of the same concentration of both oxidized and reduced peptides on the membranes of the cells. The fluorescence intensity of NPN and DiSC_3_-5 were both significantly increased by the addition of crp4oxi or crp4red, compared with the stable intensity of peptide-untreated cells. As shown in the figures, crp4s instantly (within several seconds) permeabilized the outer membrane of intact *E. coli* (Figure 2A), followed by the loss of membrane potential associated with functional disruption of the inner membrane (Figure 2B). The fluorescence intensity of crp4red was higher than that of crp4oxi in both the fluorescence measurement experiments, indicating that crp4red can more effectively perturb both the outer and inner membranes, despite its weaker activity with a 2-fold higher MBC.

Next, crp4oxi or crp4red was added to *E. coli* cultured in anaerobic environments (Figure 2C,D). The same concentration of peptides (80 μg/mL) as in the experiment in aerobic culture was used to compare the membrane damage ability under different cultivation conditions. Consistent with the fact that crp4oxi or crp4red exhibited significantly weaker antimicrobial activity under anaerobic conditions, insignificant membrane damage was detected using either fluorescent probe at this concentration. These results suggest that the membranes of *E. coli* cultured in an anaerobic environment act as protective barriers against these peptides.

### 2.3. Detection of Crp4s-Induced Oxidative Stress by Intracellular Autofluorescence Assay

The existence of other modes of action (MOA) was examined to explain the lack of concordance between their ability to directly disrupt membrane function and the intensity of their antimicrobial activity under aerobic conditions. The induction of oxidative stress and generation of reactive oxygen species (ROS) have been widely reported as the mechanisms by which antimicrobial peptides exert their antimicrobial activity under aerobic conditions [13,23,24]. ROS, which are generally considered to include superoxide, hydrogen peroxide, and hydroxyl radical, can cause several intercellular damages, such as DNA breaking, lipids peroxidation, and proteins carbonylation [25]. We investigated the association between the antimicrobial activity of crp4s and oxidative stress, as this has not been previously reported.

**Figure 3:**
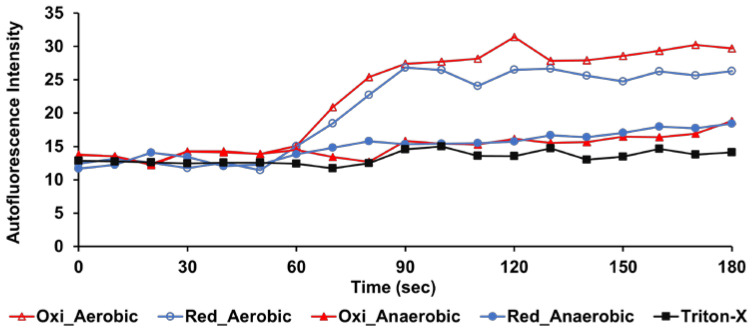
Detection of ROS generation inside *E. coli*, induced by crp4oxi and crp4red, using cellular autofluorescence experiments. The bacterial concentration was 4 × 10^7^ CFU/mL, and 80 μg/mL peptides were added 60 s after starting the measurement. One percent Triton-X was applied as a control. A representative image of three experiments is shown.

Oxidative stress was determined by measuring the intensity of the cellular autofluorescence excited at 457 nm when crp4 was added to the *E. coli* suspension. At this excitation wavelength, the autofluorescence of *E. coli* is dominated by oxidized forms of flavin adenine dinucleotide (FAD) and flavin mono nucleotide (FMN). They share a common chromophore that is non-fluorescent in its fully reduced form, but becomes weakly fluorescent when fully oxidized [26,27]. The autofluorescence intensities of *E. coli* induced by crp4s under aerobic and anaerobic conditions are shown in Figure 3. In crp4oxi and crp4red treatments, increased autofluorescence intensity was observed under aerobic conditions. The intensity of both peptides increased by approximately two-fold approximately 30 s after addition, and then reached a plateau. However, when we added crp4s to *E. coli* grown under anaerobic conditions, we did not observe an increase in the autofluorescence signal. This suggests that crp4s indeed induced oxidative stress and produced reactive oxygen species (ROS) in *E. coli*, and that oxygen was a prerequisite for the induction of such a phenomenon.

### 2.4. Evaluation of the Effects of Oxidative Stress and Its Removal on Antimicrobial Activity

For ROS induced by antimicrobial peptides to effectively trigger bacterial death, inhibition of their removal mechanism and ROS accumulation are required [27,28,29]. To evaluate the effect of ROS accumulation caused by crp4oxi and crp4red on antimicrobial activity, we conducted an experiment in which ROS accumulation was eliminated by the addition of a reducing agent. Glutathione (GSH) is a classic free radical-scavenging antioxidant that exerts its actions through several mechanisms, including direct reaction with several free radicals and thiol-disulfide interchange, that help recover the structure of altered proteins [30,31]. After confirming that GSH did not directly reduce crp4oxi to crp4red under the present conditions, we proceeded with the experiment.

**Figure 4:**
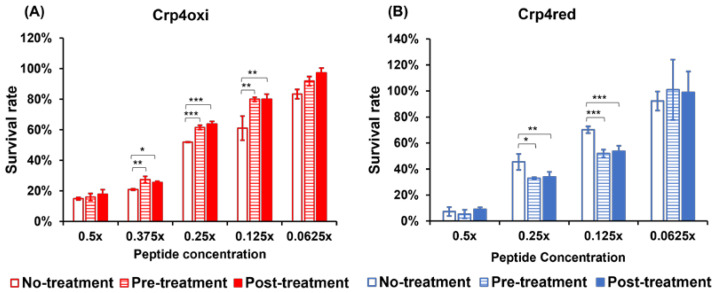
Effects of 15 mM GSH pre-treatment during *E. coli* growth, and post-treatment on the damaged *E. coli* treated by 0.0625×, 0.125×, 0.25×, 0.375×, and 0.5× MBC of crp4oxi (**A**) and crp4red (**B**). The bacterial concentration was 1 × 10^7^ CFU/ mL. Data shown reflect mean ± SEM of six or more technical replicates. Values for cells with pre- or post-treatment, and the control cells without treatment that exhibit statistically significant differences, are indicated by asterisks as follows: *, *p* < 0.01; **, *p* < 0.001; ***, *p* < 0.0001.

Sublethal damage was induced by treating *E. coli* cells with low concentrations of crp4s, and bacterial recovery following antioxidant treatment with GSH was examined. Figure 4 shows the survival rates of damaged *E. coli* induced by crp4oxi and crp4red, with and without GSH pre- and post-treatment. The antimicrobial activity of crp4oxi was inhibited by 15 mM GSH, both before and after treatment, and the survival of the bacteria eventually increased (Figure 4A). At this concentration, up to 30% of the cells could be rescued from cell death induced by 0.125 × MBC of crp4oxi. In contrast, crp4red cells exhibited reduced survival after GSH treatment (Figure 4B). These results suggest that the generation and accumulation of ROS may contribute to the antimicrobial activity of crp4oxi, but not that of crp4red.

### 2.5. Crp4 Treatment-Induced Membrane Vesicles Production under Aerobic Conditions

**Figure 5:**
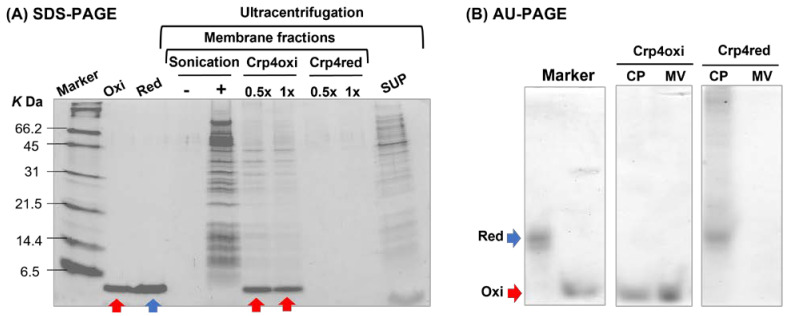
Coomassie-stained SDS-PAGE result of membrane fractions collected by ultracentrifugation assay induced by 0.5× and 1× MBC of crp4s or sonication treatment (**A**). Coomassie-stained AU-PAGE result of Cell Pellet (CP) and MV from *E. coli* after incubation with 50 μg/mL crp4s (**B**). Crp4oxi (Oxi) and crp4red (Red) were applied to show the position of peptides in PAGEs. Sonication samples were applied as bacterial membrane fractions control. The supernatant (SUP) from ultracentrifugation by sonication treatment was applied to show intracellular proteins released from cells. A representative image of three experiments is shown.

To further evaluate the effects of oxidative stress induced by crp4oxi under aerobic conditions, we examined one possible structural change in cell membranes treated with crp4oxi. Previous reports have described cases in which small membrane vesicles (MVs) enriched in peptides are produced as transporters to remove AMPs when antimicrobial peptides exert antimicrobial activity through oxidative stress [32,33]. Based on the nearly identical surface components of MVs and the bacterial membrane, MVs are often identified by the widely distributed membrane proteins and membrane lipids. Ultracentrifugation, the most common method used to separate MVs, was selected in our experiment, by which membrane fractions would form precipitates [34]. SDS-PAGE was performed on precipitates collected by ultracentrifugation to analyze bacterial membrane proteins and crp4s (Figure 5A). Sonication was used as a control because it is a strong method that destroys bacterial membranes, resulting in membrane fractions enriched in different kinds of membrane proteins, and releasing intracellular components to the supernatant (SUP) [34,35]. In the precipitate fraction by ultracentrifugation after crp4oxi treatment, a very obvious crp4oxi band was observed, along with several presumed membrane-derived protein bands. This suggested that cells might release MVs with crp4oxi as a response to the induction of oxidative stress by crp4oxi, whereas crp4red showed only bands that were similar to those in the control without peptide treatment. This suggested that MVs are not formed upon treatment with crp4red.

To further investigate the redox state of crp4s and their binding to bacterial membranes, we performed an acid urea PAGE (AU-PAGE) analysis of CP and MV when *E. coli* was exposed to higher concentrations of crp4s. AU-PAGE is a type of native PAGE that can distinguish between the different disulfide patterns of α-defensins [7]. Because crp4red lost its secondary structure and exhibited a linear structure, it migrated slightly more slowly than crp4oxi. Figure 5B clearly shows the presence of high concentrations of peptides in the MVs fraction of the crp4oxi treatment sample, but not in the crp4red-treated group. Despite the lower amount of MVs compared to the recovered CPs, crp4oxi-derived bands in MVs of comparable intensity were identified, suggesting that crp4oxi was enriched in MV. In contrast, crp4red showed a clear band at the same position as the marker crp4red only in the CP, as well as several bands with lower mobility than crp4red. As this was the result of AU-PAGE without reducing agents, these bands may have been caused by crp4 forming disulfide bonds with various cell-derived proteins.

### 2.6. Evaluation of DNA-Binding Ability of Crp4s

Next, we examined the MOA associated with the moderate antimicrobial activity (MBC > 50 μg/mL) observed with crp4red under anaerobic conditions (Figure 1D), because no direct damage-induced reduction in the membrane potential (Figure 2D) or oxidative stress-mediated MOA (Figure 4) was observed in bacteria treated with crp4red under anaerobic conditions. To explore the possibility of exerting antimicrobial activity using different MOA, we focused on the interaction with DNA, an intracellular target of antimicrobial peptides [36,37], which might cause bacterial death by interfering with gene expression and further affecting biological functions [38].

**Figure 6:**
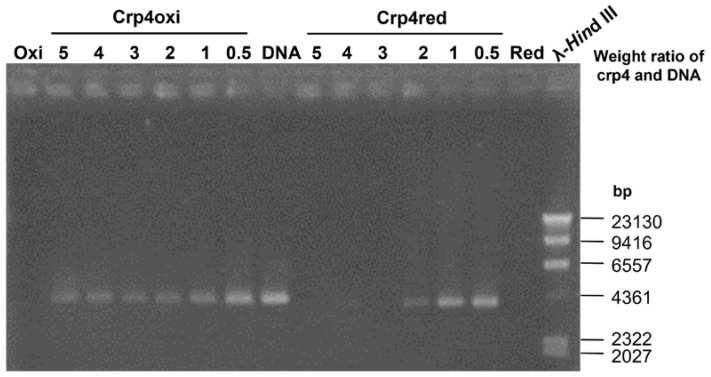
Binding ability of crp4oxi and crp4red with DNA. DNA and peptides were co-incubated for 1 h at room temperature before electrophoresis on a 0.8% (*w*/*v*) agarose gel. Two hundred ng plasmid DNA was used in one sample. One μg crp4oxi (Oxi) and crp4red (Red), and 200 ng DNA were applied as negative controls. A representative image of three experiments is shown.

The DNA-binding ability of the crp4s was evaluated using a DNA gel retardation experiment (Figure 6). At a peptide/DNA weight ratio of 0.5, nearly all DNA plasmids were able to migrate into the gel in the same way as the control. As the concentration of crp4oxi gradually increased (weight ratio of 1 to 5), the intensity of the DNA band gradually decreased; however, the DNA band did not disappear completely. In contrast, for crp4red, DNA migration was strongly and rapidly inhibited as the amount of peptide increased. When more than three times the amount of peptide by weight was added, no bands were detected, suggesting a stronger DNA-binding ability of crp4red. This might be explained by the stronger electrostatic interaction between negatively charged DNA and crp4red, which has a flexible structure with all positively charged groups fully exposed [39].

### 2.7. Structural Characterization of Crp4s by Circular Dichroism (CD) Spectroscopy

**Figure 7:**
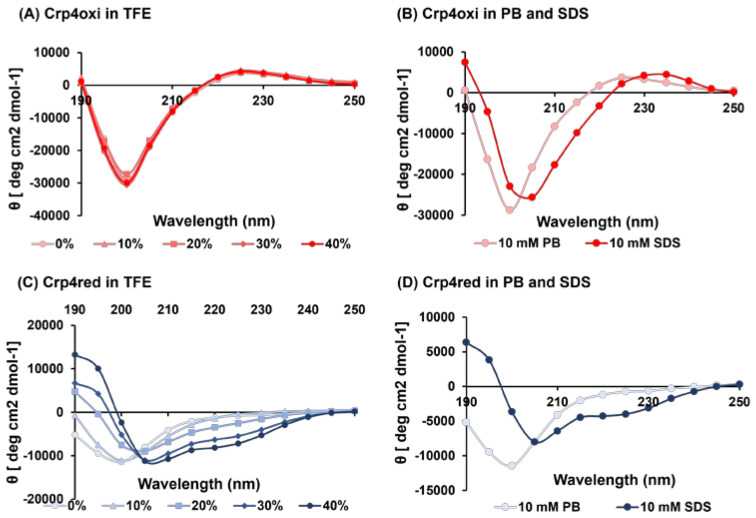
CD spectra of crp4s in various solvent conditions. Far-UV CD spectra were recorded for crp4oxi (**A**,**B**) and crp4red (**C**,**D**) in the presence of TFE, 10 mM sodium phosphate buffer (PB), and 10 mM SDS at 25 °C, and 113 μg/mL peptide was used.

To investigate the structural effects of crp4oxi and crp4red on microbes, we analyzed the proteins using circular dichroism (CD) spectroscopy (Figure 7). Consistent with previous CD analysis reports [40,41], crp4oxi in 10 mM sodium phosphate buffer (PB) showed a strong negative maximum at 200 nm, and a positive maximum at 225 nm, confirming the presence of a disulfide bond-stabilized β-sheet structure [42]. The addition of 10–40% trifluoroethanol (TFE), to confirm the structural changes in the hydrophobic environment, did not alter the spectra. Crp4oxi showed almost no structural changes at different concentrations of TFE, indicating that the structure of crp4oxi was stabilized by three disulfide bonds (Figure 7A). In the spectra of negatively charged SDS micelles in the bacterial membrane-mimicking environment (Figure 7B), no significant change in spectral shape was observed, although a slight shift to longer wavelengths, possibly due to electrostatic interactions, was observed. In contrast, crp4red in PB was considered to have a random structure, with a negative maximum around 200 nm, but an increase in the component, with negative maxima at around 208 and 222 nm, characteristic of the α-helix structure, was observed with increasing TFE concentrations (Figure 7C) [43]. Similarly, in SDS micelles, crp4red showed a CD spectrum with components characteristic of the α-helix structure (Figure 7D).

## 3. Discussion

**Figure 8:**
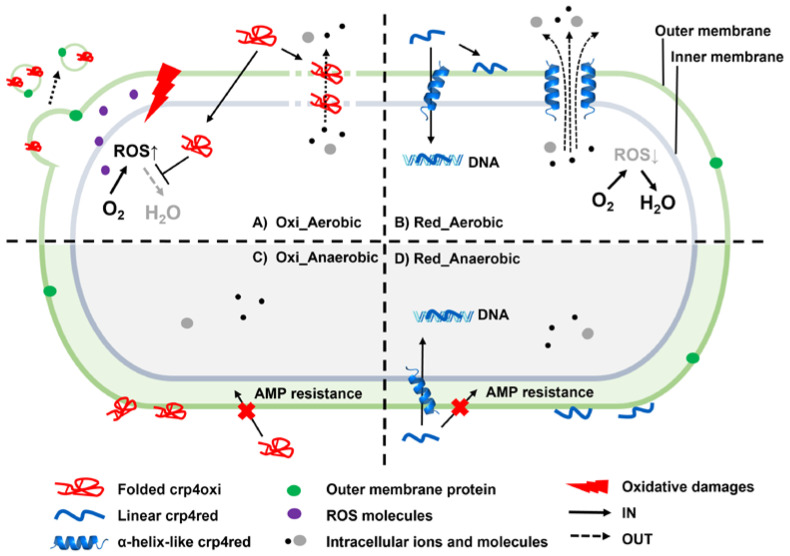
Schematic diagram for the mode of action (MOA) of crp4oxi (**A**,**C**) and crp4red (**B**,**D**) against *E. coli* under aerobic and anaerobic conditions.

In this study, we comprehensively investigated the antimicrobial activity of mouse-derived crp4, a type of α-defensin that exists in oxidized and reduced forms in vivo [9,14], against *E. coli* under aerobic and anaerobic culture conditions. The results are shown schematically in Figure 8.

The antimicrobial activity against *E. coli* under aerobic conditions, both by crp4oxi and crp4red, may be primarily due to rapid membrane damage, as previously reported (Figure 8A,B). Crp4oxi exhibits an amphiphilic structure, in which hydrophobic residues form two hydrophobic planes and positively charged residues form a hydrophilic ring because of three disulfide bonds. This property allows them to interact with lipids through electrostatic and hydration forces and distribute themselves into membrane leaflets [44]. With the permeabilization of OM and depolarization of IM, internal substances were released [40]; however, it is believed that no distinct pores were formed [45]. In contrast, crp4red, which does not have disulfide bonds, may be more efficient at disrupting membrane function by changing its conformation when bound to lipid membranes to maximize its favorable interaction with the membrane. Previous studies using our liposome leakage assay have also shown increased membrane damage caused by crp4red [40].

However, in addition to direct damage to membrane function, the results of this study suggest the possibility of ROS-mediated antimicrobial activity by crp4oxi (Figure 8A). First, we confirmed the more oxidizing condition of bacteria cells induced by crp4s under the aerobic condition through the autofluorescence experiment. Furthermore, to confirm the induction of oxidative stress with ROS production inside cells by peptide is not just a phenomenon as the secondary effect of membrane damage, but acts as the cause of antimicrobial activity, the reduced antimicrobial activity of crp4oxi in GSH-induced oxidative stress removal experiment was confirmed. Together with the observed formation of MVs containing outer membrane proteins and crp4, these data support the possibility that ROS production by crp4oxi contributed to cell death. It has been suggested that microorganisms may release MVs containing AMPs and OMPs to cope with AMP-induced oxidative stress [24,46,47]. More than half of the proteins associated with redox functions were present in the periplasm and membrane fractions, suggesting that specific inhibition of these functions by antimicrobial peptides may be important for antimicrobial activity via ROS accumulation [29]. It is possible that crp4oxi, with its stable three-dimensional structure, selectively targets redox metabolism-related enzymes in the periplasmic space after permeabilizing the outer membrane [12].

Another important finding of this study is that the antimicrobial activity of crp4s differed significantly between aerobic and anaerobic conditions, even in experiments using the same *E. coli* strain (Figure 8C,D). Interestingly, under anaerobic conditions, *E. coli* is susceptible to crp4red (MBC = 100 μg/mL), but extremely resistant to crp4oxi (MBC = 10,000 μg/mL). Under anaerobic conditions, the peptide did not induce rapid membrane permeabilization or depolarization. *E. coli* K12, as a pathogenic anaerobic bacterium, is a metabolically versatile bacteria that has three redox metabolic modes, which could transfer quickly, depending on the oxygen environment [48]. When reflecting their changes caused by the cultivation condition in the bactericidal activity assay, *E. coli* K12, under the anaerobic cultivation condition, shows higher AMP resistance ability with changed chemo-physical properties, such as a more hydrophobic surface, owing to a decrease in membrane potential, lipid/protein ratio of the membrane, and low level of ROS inside cells [49,50]. These changes in bacterial membrane properties may reduce the membrane permeation rate and delay molecular diffusion by altering the fluidity and stiffness of the plasma membrane. Therefore, crp4s may localize to the cell membrane surface and exert their slow-acting antimicrobial activity by affecting cell membrane fluidity [49]. Higher antimicrobial activity of crp4red under anaerobic conditions might be associated with the higher DNA-binding capacity of crp4red, compared to that of crp4oxi, as revealed in this study. It cannot be ruled out that the difference in the efficiency of cellular uptake by crp4red and crp4oxi through membrane permeation, and the difference in their DNA-binding capacities, may work synergistically, and contribute to the extremely large difference in the antimicrobial activity of the two under anaerobic conditions. Finally, suggested by structure change in membrane mimetic conditions by CD data, differences in the structural properties of crp4oxi and crp4red may be related to differences in the strength of their antimicrobial activities via these mechanisms.

In our study, three kinds of mode of action (MOA) of crp4s under different cultivation conditions were confirmed: membrane damage, the induction of oxidative stress with ROS production, and the DNA-binding action. The above schematical model was drawn and discussed based on the comparison of the antimicrobial activity and different abilities to cause a cellular change of each peptide under different conditions, also supported by other research [44,45]. For instance, our data suggested that crp4oxi induced weaker membrane permeabilization and DNA-binding affinity, compared with those of crp4red; however, it is believed to exhibit more potential bactericidal activity (MBC = 5 μg/mL) than crp4red (MBC = 10 μg/mL) against cells under the aerobic condition, due to the MOA of ROS production. More work is required to identify the dominant MOA of peptides under the given conditions, such as whether these effects are achieved and acted simultaneously, or whether certain MOA precede others, performed as a secondary effect, and possibly not as a killing mechanism [51,52].

Another limitation of this study is that it was limited to *E. coli* as a model bacterium. It is important to investigate the antimicrobial activity of crp4s against a wider range of commensal and non-commensal bacterial species in the intestinal tract by varying the partial pressure of oxygen in the culture. In particular, it is important to characterize crp4oxi and crp4red in vivo, including their distribution in the intestinal tract, to determine the bacterial specificity of the antimicrobial activity of crp4s in the intestinal tract. A similar investigation showed that, similar to crp4, the human-derived α-defensins HD5 and HD6 exist in both oxidized and reduced forms in the intestinal tract [9,36]. Decreased secretion or abnormal structure of defensins causes dysbiosis of the intestinal microbiota, and is associated not only with intestinal diseases, such as Crohn’s disease [7], but also with nonalcoholic steatohepatitis [53] and depression [8]. In relation to these diseases, changes in the antimicrobial activity of defensins due to their redox structure should be studied more deeply.

## 4. Materials and Methods

### 4.1. Peptides

Crp4oxi was produced by the co-expression system, and purified as described previously [54]. Briefly, the *E. coli* strain BL21(DE3) containing the pCOLA-[Cys-less HLA]-Crp4 (Novagen) vector was used in the expression of crp4 as the inclusion body. *E. coli* cells were crushed by an ultrasonic crusher (insonator 201M, KUBOTA) at 180 W for 30 min in sonication buffer (20 mM Tris-HCl pH 8.0, 1 mM EDTA), and the inclusion body was collected by centrifugation at 4300× *g* for 20 min at 4 °C. The inclusion body was then solubilized in buffer (50 mM Glycine-NaOH, 3 mM EDTA, 6 M Urea, pH 9.0) at 37 °C, 180 rpm, and refolded into crp4oxi by air oxidization. The supernatant, which contains crp4oxi, was purified by cation exchange chromatography of SP Sepharose FAST FLOW (Cytiva), equilibrated with 50 mM Glycine-NaOH pH 8.5, 3 mM EDTA. Adsorbed crp4oxi was eluted using a linear gradient of 0–1 M NaCl buffer. After overnight dialysis in 0.1% acetic acid, the crude crp4oxi sample was purified by reversed phase HPLC (RP-HPLC) using column COSMOSIL Protein-R (nacalai tesque). Elution was performed using a linear gradient of 0–50% acetonitrile with 0.1% TFA. Finally, the purified recombinant crp4oxi was lyophilized and stored at −30 °C before use. 

To obtain crp4red, 500 mM dithiothreitol (DTT) was added to 2–5 mg/mL crp4oxi solution. The solution was then incubated at room temperature. After 16 h, the reaction solution was adjusted to pH 2.0~3.0 and crp4red was purified by RP-HPLC with the same elution program, then lyophilized and stored at −30 °C before use.

Crp4oxi and crp4red were used in the full range from 0.3125 μg/mL (0.083 μM) to 10,000 μg/mL (2660 μM), and from 1.25 μg/mL (0.332 μM) to 500 μg/mL (133 μM), respectively.

### 4.2. Bacteria Cultivation

Facultative anaerobe *E. coli* K12 (ATCC 39425) was used throughout this study, which was cultured in 3% TSB (Tryptone soy broth) at 37 °C, with 180 rpm under aerobic cultivation and 50 rpm under the anaerobic condition. It took 3–3.5 h and 4.5–5 h to reach to the exponential phase (absorbance at 600 nm was 0.4) under aerobic and anaerobic cultivation conditions, respectively. An Anaero Pack system (Mitsubishi Gas Chemical Company, Tokyo, Japan) was used to maintain anaerobic growth conditions during the cultivation, incubation, and plating processes. The bacterial solution was collected by centrifugation (9300× *g*, 5 min), washed, and resuspended in an assay buffer containing 10 mM sodium phosphate buffer (pH 7.4) at different concentrations in each experiment. The concentration of bacteria was calculated by colony counting.

### 4.3. Bactericidal Activity Assays

The minimum bactericidal concentration (MBC) was determined using the broth microdilution method as described previously [54]. Briefly, cultivated *E. coli* solution, prepared using the above protocol, and crp4 solution dissolved in 10 mM sodium phosphate buffer (pH 7.4) were mixed and incubated at 37 °C for 1 h. The concentration of bacteria at this time was 1 × 10^7^ CFU/mL. Serial dilutions of crp4oxi or crp4red at a range of 20 to 0.625 μg/mL were used against *E. coli* cultivated under the aerobic condition. For cells under anaerobic cultivation treatment, 100, 500, 1000, 2500, 5000, 7500, and 10,000 μg/mL of crp4oxi and 25, 50, 100, and 500 μg/mL of crp4red were used. The final bacterial concentration was 1 × 10^7^ CFU/mL. The mixture was then diluted 1000 times and 50 μL dilution cell suspension was added to a 3% TSB medium with 1.5% agar. After culturing overnight at 37 °C, the number of colonies was compared to the control without antimicrobial peptide under aerobic and anaerobic conditions, respectively, and survival rates were calculated.

### 4.4. Assay for Peptide-Induced Membrane Damages

Membrane damages on the outer membrane (OM) and inner membrane (IM) were confirmed using methods basically as described previously [21,22].

For the OM damage assay, cultured *E. coli* cells were washed and diluted to OD600 at 0.2, with 5 mM HEPES buffer (pH 7.2) containing 20 mM glucose, by centrifugation at 2770× *g*, 25 °C, for 5 min. Afterward, peptides with the final concentration of 80 μg/mL and NPN with the final concentration of 10 μM were added 60 s after starting the measurement. The final bacterial concentration was 4 × 10^7^ CFU/mL. Changes in fluorescence were monitored continuously using a fluorescence spectrometer (F-2000 Fluorescence Spectrometer, Hitachi, Tokyo, Japan) at excitation and emission wavelengths of 355 and 408 nm, respectively. 

For the IM damage assay, *E. coli* suspension was prepared as the just mentioned method above. Subsequently, bacteria suspensions were incubated with the final concentration of 1 μM DiSC3(5) containing 0.15 mM EDTA at 37 °C, 50 rpm, for 30 min in the dark. Afterward, peptides with the final concentration of 80 μg/mL were added 60 s after starting the measurements. The final bacterial concentration was 4 × 10^7^ CFU/mL. Changes in fluorescence were monitored continuously using a fluorescence spectrometer at excitation and emission wavelengths of 622 and 670 nm, respectively.

### 4.5. Isolation of Membrane Vesicles

Membrane vesicles were isolated as described before [55]. Briefly, *E. coli* suspension prepared by the above protocol was treated for 1 h, at 37 °C with peptides. Two peptide concentrations 0.5× (2.5 and 5 μg/mL of crp4oxi and crp4red, respectively) and 1× MBC (5 and 10 μg/mL of crp4oxi and crp4red, respectively) were used. The final bacterial concentration was 4 × 10^7^ CFU/mL. Then, normal centrifugation at 4 °C, 9300× *g*, for 5 min was performed to precipitate intact or less-damaged cells as pellets (CP). Then, the supernatant was ultra-centrifugated at 4 °C, 252,000× *g*, for 30 min for obtaining precipitate as membrane fraction-containing membrane vesicles (MV).

### 4.6. Electrophoresis Assay

Acid-urea polyacrylamide gel electrophoresis (AU-PAGE) was performed as previously described [54]. Samples were analyzed using AU-PAGE on a 12.5% acrylamide gel containing 5% acetic acid and 5 M urea at 150 V. Samples were also analyzed by SDS-PAGE on a 15% acrylamide running gel and a 6% acrylamide stacking gel. Gels were stained using EzStain Aqua (ATTO Corporation, Tokyo, Japan).

### 4.7. In Vitro DNA-Binding Studies

The DNA-binding abilities of the peptides were examined using a gel retardation assay. Plasmid DNA (10 μg/mL of the pET16 plasmid) was incubated with peptides at a range concentration of 5, 10, 20, 40, and 50 μg/mL in Tris-EDTA buffer (10 mM Tris and 1 mM EDTA, pH 8.0) for 30 min at room temperature. After incubation, the peptide-DNA mixtures were subjected to electrophoresis on a 0.8% agarose gel containing ethidium bromide [36]. The gels were visualized under UV light and images were recorded using a gel documentation system (ATTO Corporation).

### 4.8. Cellular Autofluorescence Assay

The cultured *E. coli* cells were collected as the method mentioned above and washed with 10 mM phosphate buffer (pH 7.4). The final OD600 of bacteria suspension was adjusted to 0.2. Peptides with the final concentration of 80 μg/mL were added 60 s after starting the measurements. The final bacterial concentration was 4 × 10^7^ CFU/mL. Changes in fluorescence were monitored continuously using a fluorescence spectrometer (F-2000 Fluorescence Spectrometer, Hitachi) at excitation and emission wavelengths of 428 and 457 nm, respectively. Bacteria treated with 1% TritonX-100 were used as controls.

### 4.9. Antioxidant Pre-Treatment

Antioxidants were freshly prepared as mentioned below before use [27]. Stock solutions (250 mM) of glutathione were prepared in sterile distilled water, followed by filter sterilization through a 0.22-μm membrane (Millipore, Burlington, MA, USA), and added to TSB during the *E. coli* cultivation step, until the exponential growth phase (absorbance at 600 nm was 0.4~0.6). The final concentration of GSH in TSB was 15 mM. The cultivated *E. coli* solution was washed and diluted using the above protocol, and crp4, dissolved in 10 mM sodium phosphate buffer (pH 7.4), was mixed and incubated at 37 °C for 1 h. For cells treatment, 0.5×, 0.375×, 0.25×, 0.125×, and 0.0625× MBC of crp4oxi (MBC = 5 μg/mL) and crp4red (MBC = 10 μg/mL) were used. The mixture was then diluted and added to a 3% TSB medium with 1.5% agar. After culturing overnight at 37 °C, the survival rate was calculated by colony counting. The final bacterial concentration was 1 × 10^7^ CFU/mL.

### 4.10. Antioxidant Post-Treatment

Cultivated *E. coli* solution and crp4 were mixed and incubated at 37 °C for 1 h. For cells treatment, 0.5×, 0.375×, 0.25×, 0.125×, and 0.0625× MBC of crp4oxi (MBC = 5 μg/mL) and crp4red (MBC = 10 μg/mL) were used. The mixture was then diluted and added to a 3% TSB medium containing 1.5% agar and 15 mM GSH. TSB agar plates were prepared by adding stock solutions (250 mM) of glutathione to the medium before pouring the plates.

### 4.11. Circular Dichroism (CD) Spectrometry

Circular dichroism (CD) data were collected using a Jasco J 725 spectropolarimeter (Jasco, Inc., Easton, MD, USA). Spectra were collected from 250 to 190 nm and scanned at 20 nm/min at 25 °C. The bandwidth was 1.0 nm, and each data point was scanned four times under nitrogen gas. The spectra were measured in three different environments: 10 mM sodium phosphate buffer (pH 7.4), 10, 20, 30%, and 40% trifluoroethanol (TFE), and 10 mM sodium dodecyl sulfate (SDS), and 113 μg/mL peptide was used.

The mean residue ellipticity values, *θ*, were calculated using the formula below:

$$[\theta ]=\frac{\theta \;observed}{10\times n\times C\times l}$$

where *n* is the number of amino acid residues, *C* is the peptide concentration, and *l* is the optical passage length of the cell.

## 5. Conclusions

This study suggests that different antimicrobial activities and mechanisms depend on the redox-dependent structure of crp4 and the growth conditions of the target bacteria. This complexity is likely to contribute to the success and stability of this highly conserved host defense system in the constant fight against microbes.

## Data Availability

The data presented in this study are available on request from the corresponding author. The data are not publicly available due to privacy.
